# Linezolid in the Focus of Antimicrobial Resistance of *Enterococcus* Species: A Global Overview of Genomic Studies

**DOI:** 10.3390/ijms26178207

**Published:** 2025-08-24

**Authors:** Slavil Peykov, Boris Kirov, Tanya Strateva

**Affiliations:** 1 Department of Genetics, Faculty of Biology, University of Sofia “St. Kliment Ohridski”, 8 Dragan Tzankov Blvd., 1164 Sofia, Bulgaria; 2 BioInfoTech Laboratory, Sofia Tech Park, 111 Tsarigradsko Shose Blvd., 1784 Sofia, Bulgaria; 3 Department of Medical Microbiology “Corr. Mem. Prof. Ivan Mitov, MD, DMSc”, Faculty of Medicine, Medical University of Sofia, 2 Zdrave Str., 1431 Sofia, Bulgaria; 4 Department Industrial Automation, Technical University of Sofia, 1756 Sofia, Bulgaria; 5 Faculty of German Engineering Education and Industrial Management (FDIBA), Technical University of Sofia, 1756 Sofia, Bulgaria

**Keywords:** linezolid, linezolid resistance mechanisms, *Enterococcus faecium*, *Enterococcus faecalis*, whole-genome sequencing, G2576T mutation, *optrA*, *poxtA*, *cfr*

## Abstract

Linezolid (LNZ) is a synthetic oxazolidinone antibiotic that inhibits bacterial protein synthesis through binding to ribosomal RNA, also preventing the assembly of the initiation complex during translation. It is one of the last-line therapeutic options for serious infections caused by problematic Gram-positive pathogens, including vancomycin-resistant and multidrug-resistant *Enterococcus* species. Data from recent large-scale studies show a 2.5-fold increase in the prevalence of clinical LNZ-resistant enterococci (LRE) over the past decade with a global detection rate of 1.1% for LNZ-resistant *E. faecium* (LREfm) and 2.2% for LNZ-resistant *E. faecalis* (LREfs). Most reported cases have originated from China, followed by South Korea and the United States. LREfm typically belongs to the high-risk clonal complex 17, whereas LREfs demonstrates a heterogeneous population structure. Mutations in the 23S rRNA and ribosomal proteins, as well as acquired resistance genes such as *cfr*, *optrA*, and *poxtA* are involved in the development of LNZ resistance among enterococci. Whole-genome sequencing (WGS) has been recognized as a gold standard for identifying the underlying molecular mechanisms. It exposes that numerous LRE isolates possess multiple LNZ resistance determinants and mutations, further complicating the treatment strategies. The present review article summarizes all known mutational and non-mutational LNZ resistance mechanisms and presents a global overview of WGS-based studies with emphasis on resistome analysis of clinical LREfs and LREfm isolates published in the literature during the period 2014–2025.

## 1. Introduction

Linezolid (LNZ) is a synthetic antibiotic drug belonging to the oxazolidinone class. As a novel molecule, unrelated to existing drug derivatives at the time of its market introduction in 2000, it remains one of the few truly innovative antibiotics developed in recent decades. LNZ inhibits protein synthesis through binding to ribosomal RNA (rRNA), also preventing the assembly of the initiation complex during translation. High-resolution structural analysis has revealed that it binds to specific nucleotides of the 23S rRNA within a deep cleft of the 50S ribosomal subunit [1]. Notably, similar to the phenicol antibiotic chloramphenicol, LNZ can induce mitochondrial toxicity by off-target inhibition of human mitochondrial protein synthesis through its binding to mitochondrial ribosomes [2]. The inability of mitoribosomes to synthesize essential proteins of the mitochondrial electron transport chain, which are normally produced within these organelles, can lead to severe complications, including lactic acidosis, myelosuppression, and peripheral neuropathy [3], particularly during a prolonged course of treatment [4,5,6,7].

LNZ exhibits potent in vitro activity against a wide range of Gram-positive bacteria including streptococci, vancomycin-sensitive and vancomycin-resistant enterococci (VRE), coagulase-negative staphylococci, methicillin-sensitive *Staphylococcus aureus*, methicillin-resistant *S. aureus* (MRSA) [8], as well as species of *Bacillus* [9], *Corynebacterium* [10], and *Listeria monocytogenes* [11]. On the other hand, it shows low activity towards Gram-negative pathogenic bacteria since these organisms possess intrinsic resistance mediated by efflux pumps that expel the antibiotic more rapidly than it can accumulate within the cell [12]. LNZ has been approved by the U.S. Food and Drug Administration (FDA) for the treatment of hospital-acquired pneumonia caused by *S. aureus* (including MRSA) and *Streptococcus pneumoniae*, as well as community-acquired pneumonia due to *S. pneumoniae*. Additionally, LNZ is indicated for infections caused by vancomycin-resistant *Enterococcus faecium* (VREFm), complicated Gram-positive skin and skin structure infections, including diabetic foot infections, and pneumococcal meningitis caused by penicillin-resistant *S. pneumoniae* [1,13,14]. It has also been repurposed for the treatment of drug-resistant and complicated multidrug-resistant tuberculosis [15] and is well tolerated in pediatric patients infected with *Mycobacterium* spp. [16] as well as other Gram-positive pathogens [17].

LNZ is especially prominent in the treatment of severe and life-threatening infections caused by *Enterococcus* species (spp.). Enterococci, primarily *Enterococcus faecalis* and *E. faecium*, are ubiquitous Gram-positive bacteria with an ambivalent nature. They predominantly colonize the intestinal tract of insects, reptiles, birds and mammals, including humans, where they serve as an essential component of the intestinal microbiota [18]. Their presence can also be detected in other anatomical sites of the human body, such as the oral cavity [19] and vagina [20]. Additionally, enterococci have been isolated from soil, water [21], and various fermented foods [22], indicating that they are important elements in the microbial ecology of environmental habitats. On the other hand, enterococci are also recognized as major opportunistic pathogens responsible for healthcare-associated and community acquired infections worldwide, including urinary tract infections, abdominal and biliary tract infections, endocarditis, bacteremia, sepsis, burn and surgical site wound infections, among others [23]. Because of attributes such as the capability to grow over a wide range of temperatures and at various pH levels, biofilm formation, withstand desiccation, and grow in the presence of 6.5% NaCl and 40% bile salts [24,25], enterococci are extremely well suited to survive, linger and disseminate in various hostile environments. These adaptations are further reinforced by their intrinsic resistance to several antimicrobials, such as cephalosporins, macrolides, clindamycin, and aminoglycosides (low-level), as well as their ability to acquire genetic determinants that confer resistance to multiple antimicrobial agents [26]. In addition, the genomes of clinical isolates harbor a substantial number of mobile genetic elements [27], along with numerous plasmids [28], including linear forms [29], which have been shown to carry antimicrobial resistance-related genes and mediate horizontal gene transfer. Moreover, *E. faecium* is a member of the “ESKAPE” group, which also includes *S. aureus*, *Klebsiella pneumoniae*, *Acinetobacter baumannii*, *Pseudomonas aeruginosa*, and *Enterobacter* spp. These bacteria are capable of evading the antimicrobial effects of numerous antibiotics and are responsible for the majority of nosocomial infections worldwide [30].

Among enterococci, VRE pose a particularly serious threat, with a global mortality rate ranging between 60% and 70% [31]. In 2017, the World Health Organization (WHO) placed VREfm on the “Global Priority List of Antibiotic-Resistant Bacteria” as a high-priority pathogen, thereby emphasizing the urgent need for effective treatment strategies [32]. This designation was reaffirmed in the most recent 2024 edition of the list [33]. The widespread global emergence of VRE has created a critical need for alternative therapeutic agents such as LNZ, daptomycin, and tigecycline, which are considered last-resort options for treating VRE infections [26]. Nevertheless, LNZ-resistant enterococci (LRE) are now found worldwide. They have been reported on all continents except Antarctica, with prevalence data available from 28 countries, most reports originating from China, followed by South Korea and the United States [34]. LRE-associated urinary tract and bloodstream infections have been identified as the most common clinical manifestations [35]. Furthermore, patients with LRE infections exhibited a significantly higher risk of in-hospital mortality (OR 9.3; 95% CI: 1.8–51.2) compared to those with infections caused by LNZ-susceptible enterococci [36].

In the face of high levels of global antimicrobial resistance (AMR), the application of innovative approaches to its study and control is needed. Whole-genome sequencing (WGS) has emerged as a cost-effective, high-resolution technique for genome analysis, offering critical insights into AMR genes, genomic mutations, and the evolutionary dynamics of AMR in various bacterial pathogens. It can also enable the prediction of AMR phenotypes, further enhancing its value for surveillance and clinical applications [37,38]. Moreover, genome data can, in certain contexts, be further complemented by metagenomic and metatranscriptomic surveys, employing either read-based or assembly-driven strategies. Such integrative approaches provide a broader perspective on the diversity and functional activity of AMR-associated genes within pathogenic populations, while simultaneously obviating the need for prior cultivation. In addition, they enable the detection of potential perturbations in the resident microbiota at the infection site, thereby offering critical insights into host–microbe interactions under antimicrobial selective pressure [39].

The present review article provides a global overview of WGS-based studies that have focused on LNZ resistance mechanisms, in clinical *Enterococcus* spp. isolates during the period 2014–2025. Its principal novelty lies in detailed resistome analysis, distinguishing it from the broader literature on oxazolidinone resistance in these bacteria.

## 2. Literature Search and Inclusion Criteria

The emphasis of this narrative review is placed specifically on publications that include at least one sequenced genome of *Enterococcus* strain identified as a causative agent of human infection. Studies analyzing genomes of LNZ-resistant isolates derived from environmental sources, such as wastewater, sewage, livestock, slaughterhouses, food products, or animals, were excluded. Similarly, studies focusing exclusively on colonizing isolates found through routine screening programs were also omitted.

All publications included in the present review were retrieved from the PubMed database in July 2025. The search was conducted applying the key words “linezolid resistant”, “linezolid resistance”, “*Enterococcus faecalis*”, “*Enterococcus faecium*”, and “genome” in appropriate combinations. The final queries used were ((*Enterococcus faecium*) *AND* (*genome*)) *AND* ((*linezolid resistant*) *OR* (*linezolid resistance*)) and ((*Enterococcus faecalis*) *AND* (*genome*)) *AND* ((*linezolid resistant*) *OR* (*linezolid resistance*)), yielding 175 and 161 articles, respectively. The retrieving process was not restricted by language, time, or country. Next, all abstracts, as well as the “Materials and methods” sections of the identified articles, were subsequently manually reviewed to identify studies reporting sequenced genomes of LNZ-resistant *Enterococcus* strains that were confirmed as causative agents of infections in humans. As the earliest publication reporting a sequenced genome of LRE was published in 2014, this year was designated as the starting point of our study.

This review relied exclusively on PubMed for literature retrieval. Although PubMed offers broad coverage of biomedical research, this may have resulted in the exclusion of studies published in sources not indexed in the database, such as regional journals or conference proceedings. In addition, a time-lag bias is possible, as recently published articles may not yet have been indexed at the time of the search.

## 3. LNZ Resistance in Enterococci

### 3.1. Historical Perspectives, Global Prevalence, Clonal Spread, and Mechanisms

Upon the implementation of LNZ, studies on cross-resistance with other protein synthesis inhibitors, including chloramphenicol, macrolides, lincosamides, streptogramins, aminoglycosides, and tetracyclines, demonstrated that its activity remains unaffected by modifying enzymes, efflux mechanisms, or target modification and protection by ribosomal methylases [40]. Moreover, early attempts to induce resistance in different Gram-positive isolates using gradient plates yielded no resistant colonies, suggesting that the occurrence of resistant variants is likely to be less than 1 per 10^8^ bacteria [41]. These findings, together with the unique mechanism of action of oxazolidinones, initially fostered optimism that resistance would not emerge as a significant concern for an extended period. Indeed, it was not observed in staphylococci or streptococci during the initial clinical trials [42]. However, the 1999 Linezolid Compassionate Use Program revealed that VREfm isolates from two infected patients who received LNZ treatment developed resistance to the antibiotic due to mutations in multiple copies of their 23S rDNA loci [43]. Despite this early indication of the remarkable capacity of enterococci to acquire resistance, even against drugs like oxazolidinones, the emergence of resistant clinical isolates remained sporadic and isolated during the initial years of application. Data from the two largest surveillance programs for the period 2002–2014 show low proportions of LNZ-resistant enterococci (LRE), with rates of 0.22% (21/9417; ZAAPS) and 0.78% (67/8604; LEADER), respectively [35]. However, the current situation is becoming increasingly alarming, as evidenced by a recent systematic review and meta-analysis assessing resistance to the last-resort antibiotics daptomycin, tigecycline, and LNZ among clinical enterococci worldwide. It analyzed more than 120,000 *Enterococcus* isolates from 43 countries, reporting detection rates of 1.1% (95% CI: 0.3–1.9) for LNZ-resistant *E. faecium* (LREfm) and 2.2% (95% CI: 1.5–2.8) for LNZ-resistant *E. faecalis* (LREfs) [26]. Additional confirmation of reported data comes from another large-scale global systematic study focused on LNZ resistance in enterococci, which encompassed data from 84 countries and over 157,000 isolates. The study reported a pooled prevalence of LRE of 1.9% (95% CI: 1.3–2.8%) in human isolates and 6.3% (95% CI: 3.1–12.3%) in animal isolates [34]. Interestingly, in both meta-analyses *E. faecalis* was pointed as the predominant LNZ-resistant species, which contrasts sharply with earlier ZAAPS and LEADER data were LREfm was dominant. This shift is largely attributed to the rapid dissemination of the plasmid-borne resistance determinant *optrA*, which has spread extensively among *E. faecalis* isolates in recent years.

Several studies have reported the nosocomial spread of LRE [35]. Two reports from Germany focusing on LREfm identified clonal complex 17 (CC17) as an endemic clone, based on multilocus sequence typing (MLST) [44,45]. This finding is supported by studies conducted by Nasir et al. in Pakistan, where whole-genome sequencing-based MLST analysis revealed the circulation of CC17 sequence types known for their outbreak potential [46]. Furthermore, a *vanA*-carrying vancomycin-resistant LREfm clone belonging to sequence type (ST) 117 was implicated in a documented outbreak in the United States [47]. This observation is of particular concern, as ST117 is part of clonal lineage 78, which is well recognized worldwide for its association with invasive VREfm infections [48]. Another member of lineage 78, ST203, was identified as the most frequently detected ST among LREfm strains exhibiting concomitant resistance to vancomycin in Belgium [49]. Notably, epidemiological data from the past decade indicate a marked clonal shift, with lineage 78 progressively displacing lineages 17 and 18 across multiple countries [48].

In contrast, LREfs typically exhibit a heterogeneous population structure, with multiple sequence types identified during nosocomial transmission events [35]. Nevertheless, the study by Mortelé et al., which investigated the epidemiology and genetic diversity of clinical LRE isolates in Belgium from 2013 to 2021, identified ST480 as the most frequently detected sequence type among LREfs strains, accounting for 42 of 63 typed isolates (67%) [49]. While all LREfs isolates analyzed in this work retained susceptibility to vancomycin, a large-scale investigation from China encompassing 13,556 clinical *E. faecalis* isolates reported the concerning observation that the prevalence of LNZ resistance among VREfs isolates was 24.44%, a rate significantly higher than that observed in vancomycin-susceptible *E. faecalis* [50]. Moreover, there have been reports of *E. faecalis* isolates harboring plasmids co-carrying *vanA* and *optrA* from both the United States and Italy [51,52].

Alongside the numerous surveillance programs monitoring LNZ resistance in enterococci, extensive research has been conducted to elucidate the underlying resistance mechanisms in *Enterococcus* spp. These studies have identified several contributing factors, including mutations in the 23S rRNA genes and amino acid substitutions in key ribosomal proteins (L3, L4, and/or L22). Additionally, resistance may arise through the acquisition of broad-spectrum resistance determinants, including genes encoding 23S rRNA methyltransferases (*cfr*) and ribosomal protection proteins (*optrA* and *poxtA*) [26]. The following subsections present an overview of these resistance mechanisms, along with a summary of publications that include sequenced LREfm and/or LREfs genomes where they have been identified.

### 3.2. Mutational Mechanisms Conferring LNZ Resistance in Enterococci

LNZ exerts its antibacterial effect by binding to the 50S subunit of the prokaryotic ribosome, thereby preventing its association with messenger RNA (mRNA), the 30S subunit, fMet-tRNA, and initiation factors 2 and 3 [53]. Such inhibition blocks the formation of a functional initiation complex, ultimately halting the translation at the earliest stage. This mechanism differs from that of other antibiotics, such as chloramphenicol, macrolides, lincosamides, and tetracyclines, which also bind to the ribosome but allow translation to initiate before disrupting peptide elongation at a later point. It is also thought to provide unique advantages to LNZ, particularly its effectiveness in suppressing the production of certain streptococcal and staphylococcal virulence factors, such as hemolysins, coagulase, and protein A [54]. Nevertheless, its binding to the bottom of the cleft at the center of the 50S ribosomal subunit, as evidenced by numerous crystal structures of LNZ–50S ribosomal subunit complexes from various bacteria, relies on extensive interactions with multiple phylogenetically conserved nucleotides in the peptidyl transferase loop of domain V of 23S rRNA.

The most direct approach to protecting the ribosome from LNZ is through modifications to its binding pocket. These modifications fall into two categories: 23S rRNA mutations and mutations in genes encoding for ribosomal proteins located near the peptidyl transferase center. They will be discussed in the following subsections.

#### 3.2.1. 23S rRNA Mutations

**Figure 1:**
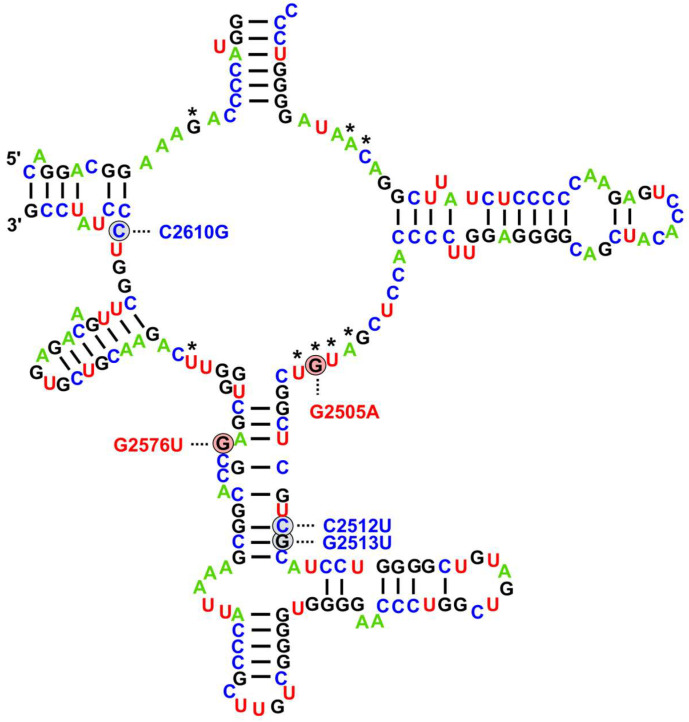
Linezolid resistance-associated mutations in enterococci affecting the peptidyl transferase loop of domain V in 23S rRNA. Base positions are referenced according to the *Escherichia coli* numbering. The nucleotides that contact directly with the linezolid are denoted by an asterisk. Mutations conferring resistance to linezolid are color-coded based on their significance: red for common variants with a well-established role, and blue for recently identified variants associated with advancements in sequencing technologies. Mutation positions are taken from Turner et al. [56].

The described interactions with multiple conserved nucleotides in the binding pocket of LNZ indicate that mutations in that region can block its binding, which, for a long time, remained the only known mechanism of resistance [55]. Interestingly, studies on in vitro-selected resistant strains have shown that such mutations can affect not only the universally conserved nucleotides directly involved in the binding but also more distal positions. Moreover, the identified variants exhibit species-specific patterns, with minimal overlap between different bacteria [55]. Subsequently, these findings were also confirmed in naturally occurring resistant isolates. The identified 23S rDNA mutations in LREfm and LREfs are present in Figure 1 [56].

As shown in the figure, two mutations in enterococci, G2576U and G2505A, are strongly associated with LNZ resistance. While the G2505A variant affects a nucleotide within the LNZ binding pocket, G2576U does not directly interact with the antibiotic. Despite that, a study investigating both variants introduced into a *Mycobacterium smegmatis* strain with a single rRNA operon demonstrated that the G2505A mutation resulted in an 8-fold increase in the minimum inhibitory concentration (MIC) (from 2 to 16 mg/L), while the G2576U mutation led to a 32-fold increase (from 2 to 64 mg/L)—the highest increase among all variants studied [57]. Notably, the G2576U variant is also linked to the most significant reduction in the growth rate of the modified *M. smegmatis* cells, with doubling times increasing by a factor of 2.6. This highlights that the level of resistance conferred by a specific 23S rDNA variant is not simply determined by the nucleotide’s proximity to the antibiotic in the spatial structure of the binding site. In fact, mutations at more distal nucleotides, which do not interact directly with LNZ, can still lead to high-level resistance. These findings have been consistently validated by numerous studies on LNZ-resistant Gram-positive pathogens, highlighting G2576U as the most critical single mutation linked to the resistance phenotype [55]. It is also the most detected 23S mutation in clinical LRE, where its presence alone can result in MICs ≥ 256 mg/L, depending on the number of affected 23S rDNA loci.

Ironically, while the presence of multiple rRNA operons in enterococci was initially thought to hinder LNZ resistance development through spontaneous mutations, it now presents a major obstacle in detecting 23S rDNA variants using whole-genome sequencing (WGS). The adoption of WGS as the preferred tool for resistome studies traces back to the first fully sequenced genome of a self-replicating, free-living bacterium (*Haemophilus influenzae Rd*) where analysis of the assembled sequence revealed several genetic determinants associated with antibiotic resistance [58]. Today, WGS is globally recognized as the gold standard for detecting resistance mechanisms in clinical microbiology [59]. The most widely used sequencing platforms for bacterial analysis rely on second-generation sequencing methods based on sequencing by synthesis [60]. These technologies are preferred due to their superior cost-efficiency per analysis compared to other next-generation sequencing (NGS) approaches. However, they generate millions to billions of short reads, which inherently have higher error rates than classical Sanger sequencing. Subsequently, these short reads can be utilized either for de novo assembly of draft genome sequences or for mapping against the genome of a reference strain to identify sequence variations [61]. In bacterial genomics, the first option is far more commonly used, primarily due to the relatively small size of bacterial genomes (compared to those of higher eukaryotes), their haploidy, and the low proportion of repetitive regions. These characteristics enable assembly software tools to perform the process efficiently and quickly, with relatively low computational requirements. Next, resistome analysis is conducted on the assembled genome sequence using various software tools, such as ResFinder, among others [62]. The major issue with this workflow arises during the assembly step, where de novo assemblers collapse multicopy regions with nearly identical sequences, significantly longer than the sequencing reads, into a single copy, with the most frequent variants represented as a consensus. Unfortunately, the 23S rRNA loci fall into this category, meaning that mutations affecting less than 50% of these genes can easily be overlooked. This phenomenon has been described for genetic variants in the 23S rRNA responsible for macrolide-lincosamide-streptogramin (MLS) resistance in *Neisseria gonorrhoeae* [63]. Considering that *E. faecium* genomes typically harbor an average of six 23S rRNA loci, while *E. faecalis* genomes have four, it is evident that analyzing the assembled genome sequence in these pathogens is not an effective approach for detecting LNZ-resistance-related rRNA mutations. This issue became apparent with the emergence of the first clinical LRE isolates, leading to the use of amplicon-based pyrosequencing followed by read mapping to rapidly detect and estimate the number of 23S rRNA genes with the G2576T mutation. The method demonstrated full concordance with the PCR-restriction fragment length polymorphism (RFLP) approach using the *NheI* enzyme for detecting isolates heterozygous for this mutation [64]. Since then, sequencing has been widely implemented in numerous studies analyzing the 23S rRNA loci in LRE using WGS, primarily with the Illumina platform, which has become the standard over the years. The NGS-based analysis outperforms the alternatives in the face of qPCR and PCR-RFLP by enabling the discovery of novel variants in the 23S rRNA of LRE. This raises additional issues, as such variants are often of unclear significance. To assess their potential involvement in LNZ-resistance mechanisms in enterococci, it is essential to determine whether they have already been implicated in resistance phenotypes in other widely spread Gram-positive pathogens, such as staphylococci or streptococci. Although it may seem like a straightforward task, such comparisons are complicated and prone to errors due to the widely accepted practice of numbering positions in the 23S rRNA according to the *E. coli* sequence, as well as the fact that 23S rRNA genes vary in length, nucleotide sequence, and copy number among bacterial species. To streamline the process, Beukers et al. provided valuable recommendations and guidelines for determining LNZ resistance from WGS data [65].

Hassman et al. took this a step further by developing and validating a web-based tool for detecting 23S rRNA mutations (G2576T and G2505A) and other acquired LNZ resistance determinants in NGS data from enterococci [66]. It is extensively utilized in many of the WGS-based studies of LRE discussed in this review. Other researchers opt for general short-read aligners, such as Bowtie2 [67] or BWA [68], to map sequencing reads to the reference 23S rRNA sequence, achieving comparable while providing greater flexibility for integration into multipurpose processing pipelines.

The following subsections present studies conducted to date on WGS-based detection of 23S rRNA mutations associated with LNZ resistance in clinical LRE. A summary of the data from these studies is provided in Table 1.

Our literature review identified a total of 32 studies that have reported the sequenced genome of at least one clinical LNZ-resistant *Enterococcus* spp. isolate. Among them, the G2576U variant is by far the most frequently detected, appearing in 28 studies. Other 23S rRNA variants were observed sporadically, often in combination with additional resistance mechanisms, such as the G2576U mutation itself [70] or a co-occurrence of the *optrA* determinant with a missense mutation in the L4 ribosomal protein [71]. Notably, both these isolates originated from Austria, with a partially overlapping time frame, despite one being LREfm and the other LREfs. Particularly intriguing is the study by *Bao* et al., which describes the genomes of two LREfs isolates carrying novel mutations in the domain V loop of the 23S rRNA locus [76]. These isolates are of special interest due to their unusual origin. They were recovered from two unrelated patients diagnosed with infective endocarditis following renal transplantation. Unfortunately, in both cases, the infections had a fatal outcome.

A total of 22 studies have reported sequenced genomes of LREfm isolates carrying 23S rRNA mutations, while the corresponding number for LREfs is eight. Notably, only one study from Ireland presented genomes of both species [85]. The number of sequenced genomes per study ranged from 1 to 96. However, it is important to interpret the highest value, reported in a study from Germany, with caution, as many of these isolates were classified as colonizers rather than confirmed causative agents of infections, despite being recovered from clinical settings [81].

The majority of studies were conducted in Asia, Europe, and North America, with single reports from Brazil (South America) and Australia. No genomes with 23S rRNA mutations have been reported from Africa. Only three countries have more than two studies in this category: China, with three studies (all focused on LREfs genomes); Germany, also with three studies; and the USA, with eight studies (seven on LREfm genomes and one on LREfs). These findings support the hypothesis that LNZ resistance mechanisms in enterococci exhibit geographical variation [95].

Another notable observation is the variation in the number of 23S rRNA loci affected by the G2576U variant, with reported values ranging from 1 to 5, sometimes even within a single study [85]. Early research demonstrated a clear correlation between the number of 23S rRNA genes carrying G2576U and the level of LNZ resistance observed in clinical *E. faecium* and *E. faecalis* isolates [97]. The articles reviewed by us show a reverse trend of lack of clear correlation which is most visible in the interesting work of Egan et al. [85]. The authors present 18 LREfm isolates with sequenced genomes, each exhibiting varying copy numbers of the G2576U variant as the sole resistance mechanism. These are summarized in Table 2.

All isolates were recovered from a single country (Ireland) over a three-year period, with many sharing the same sequence type (ST) and number of G2576U-affected loci. However, their minimum inhibitory concentration (MIC) values vary by up to 16-fold (e.g., isolates 4 and 5 in Table 2). Notably, some isolates with only two affected copies exhibit significantly higher levels of LNZ resistance than those with four affected copies (e.g., isolates 1 and 7 in Table 2).

All of this leads to the conclusion that the copy number of the G2576U variant is a poor predictor of LNZ resistance levels in more heterogeneous LRE populations. Even more concerning is the fact that isolates with only two affected copies can exhibit the highest levels of LNZ resistance. When only two copies are mutated, the remaining four wild-type 23S rRNA loci can mask their presence in the assembled draft genome sequence. Indeed, two studies from the USA explicitly state that they could detect the variant only in sequencing reads, as it was absent from the final assemblies [47,90]. This suggests that genome-based analyses may only reveal “the tip of the iceberg” when it comes to G2576U-mediated LNZ resistance in enterococci, as this mutation is far more likely to arise spontaneously in fewer than half of the 23S rRNA loci. Even if a low-copy-number G2576U mutation does not result in a significant MIC increase in the background of a given isolate, its presence is far from benign. Recombination between 23S rRNA alleles has been shown to significantly increase the number of mutated copies in *E. faecalis* under selection, suggesting that LNZ-resistant isolates may emerge much faster than initially anticipated [98]. Not only patients undergoing prolonged treatment are at risk, but also those with shorter exposure to LNZ. The idea for “hidden by the assembly” G2576U variants is further supported by the very low number of LREfs genomes with that mutation found in GenBank, as reported by Strateva et al. [73]. The key takeaway from these findings is that when reporting LRE genomes, it is essential to upload the raw sequencing reads to a public repository alongside the assembled genome sequence. This ensures that future analyses can accurately assess resistance-associated mutations, even when they are present in only a subset of rRNA loci.

#### 3.2.2. Mutations in Ribosomal Protein Genes

Undoubtedly, 23S rRNA mutations are the primary driver of LNZ resistance through alterations in ribosome structure, but they are not the only factor. Ribosomal proteins, the second type of biomacromolecules within the ribosome, can also play a role. Although the LNZ binding pocket consists entirely of nucleotides from the 23S rRNA, mutations in ribosomal proteins L3 and L4, which border the peptidyl transferase center where that pocket is located, have also been associated with elevated LNZ MIC levels [99]. Notably, this increase is generally modest, as evidenced by a study in which all LRE isolates harboring only missense variants in conserved regions of their ribosomal proteins exhibited reduced susceptibility to LNZ (MICs of 4–8 mg/L) rather than true resistance [100]. Of course, such findings should not be regarded with excessive reassurance, as sequence variations in the L3 and L4 ribosomal proteins can act synergistically with other mechanisms, leading to highly resistant isolates. A key positive point is that, unlike the 23S rRNA loci, ribosomal protein genes in bacteria are typically single-copy, eliminating the above-mentioned complications in sequencing read processing.

A summary of the data from WGS studies that have identified missense mutations in the genes for the ribosomal proteins L3 and L4 is presented in Table 3.

In contrast to genomes harboring LNZ-related 23S rRNA mutations, only nine published studies have reported LRE genomes with identified mutations in ribosomal protein genes. Moreover, the number of detected variants is significantly lower than that of genomes carrying the G2576U mutation alone. This discrepancy can be attributed not only to the lesser contribution of L3 and L4 protein variations to LNZ resistance but also to study design biases. A notable example is the genomes presented from the SENTRY Antimicrobial Surveillance Program [105], where, despite detecting isolates with additional L4 mutations via amplicon sequencing, only those positive for *optrA* underwent WGS. Such biases in LRE selection for sequencing may lead to an underestimation of the significance of ribosomal protein mutations, particularly since no alternative molecular methods beyond sequencing have been widely adopted for their detection.

The data presented clearly show that the most frequently detected variant is the amino acid substitution F101L in the L4 ribosomal protein, which was identified in 26 LRE isolates across three different studies. However, in all instances, this variant was accompanied by other LNZ resistance mechanisms in the respective strains. Additionally, Bender et al. identified this variant in the LNZ-susceptible laboratory *E. faecalis* OG1RF strain, further questioning its significance in the resistance phenotype [82]. Another L4 mutation detected, a 71G72 insertion, is more intriguing, as it is the only variant detected in the genome of the investigated LREfs [102]. Notably, the isolate was recovered from the urinary tract of a patient with multidrug-resistant tuberculosis who had received LNZ as part of their treatment for 24 months. Moreover, Bender et al. identified comparable glycine insertion at position 71 in the L4 protein of LREfm in two isolates, both of which were also positive for *cfr(B)* [83]. These circumstances suggest a high probability that this mutation is related to LNZ resistance.

The only variant identified in the L3 ribosomal protein is the T150A amino acid substitution. It was found in the genome of a vancomycin-resistant LREfm from Ireland [86]. Unfortunately, its contribution to the resistance phenotype of this isolate is difficult to assess, as it coexists with the G2576U variant affecting four of the 23S rRNA loci, as well as the *optrA* and *cfr* resistance determinants. However, the accumulation of LNZ resistance determinants, particularly in a VRE isolate, warrants attention. Moreover, the same variant was also identified in 6 *optrA*-positive LREfs from Scotland [103].

Remarkably, two studies identified mutations in the L22 ribosomal protein, which are very rare in enterococci, despite being described in other LNZ-resistant Gram-positive pathogens. The first variant, found in the genome of LREfs from Austria, does not result in an amino acid substitution and coexists with the *optrA* and 23S rRNA mutations [71]. Due to this, it is unlikely to contribute to LNZ resistance. In contrast, the Ser77Thr missense mutation in the L22 ribosomal protein of *E. faecium* from China is particularly noteworthy, as WGS analysis revealed this variant as the sole plausible explanation for the observed LNZ resistance (MIC = 4 mg/L) [101].

**Figure 2:**
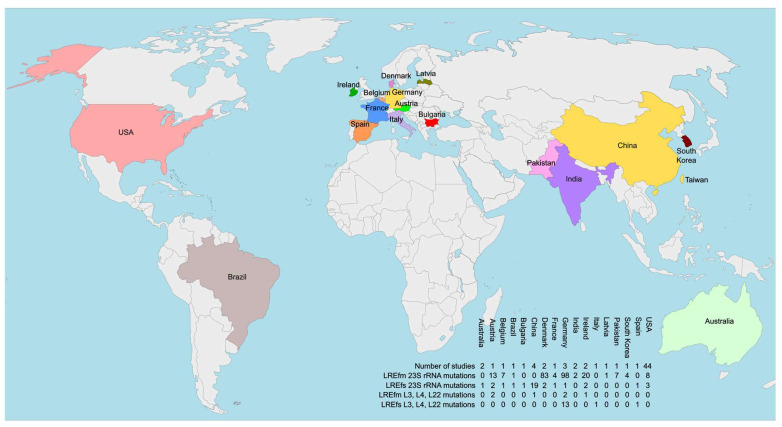
Geographical distribution of studies reporting sequenced genomes of linezolid-resistant *E. faecium* and/or *E. faecalis* strains harboring 23S rRNA mutations and/or mutations in genes encoding ribosomal proteins L3, L4, and L22.

Another notable aspect of the studies identifying ribosomal protein mutations in LRE is their geographic distribution. Except for the SENTRY program, all studies originate from Europe, excluding the Chinese isolate with a L22 mutation. A combined map displaying all studies that have identified mutations in ribosomal structural components (rRNA and proteins) along with their geographical locations is presented in Figure 2.

### 3.3. Non-Mutational Mechanisms Conferring LNZ Resistance in Enterococci

To date, all identified LNZ resistance mechanisms in enterococci prevent the binding of the antibiotic to its binding pocket in the ribosomal peptidyl transferase center. The most direct ones, discussed in the previous subsection, involve alterations in ribosomal components—specifically, the 23S rRNA and all ribosomal proteins located near the binding pocket. They rely on spontaneous mutations that are not transferable via horizontal gene transfer.

In contrast, another class of resistance mechanisms indirectly prevents LNZ from binding to its target without altering the genetically encoded ribosomal structure. This group relies on the activity of proteins encoded by three main types of determinants—*cfr*, *optrA*, and *poxtA* [106]. A notable characteristic of these genes is their association with a wide range of mobile genetic elements including IS*1216*, Tn*6218*, and Tn*1546* among others, which facilitates horizontal gene transfer and significantly increases the risk of widespread dissemination [107]. Since these determinants are not part of the core enterococcal genome and are significantly larger than point mutations, their detection via WGS is straightforward, even when using a de novo assembly approach. Many studies employ classical PCR-based screening of isolates before sequencing to select specific LRE subcategories for WGS-based resistome analysis. Primer combinations have been designed to simultaneously detect all acquired LNZ resistance gene types in clinical enterococcal isolates [108]. Such pre-selection should be considered when interpreting results, as it may introduce bias by favouring isolates with coexisting resistance mechanisms.

While detecting acquired LNZ resistance determinants in *Enterococcus* spp. isolates is simple and reliable, determining their precise location using only Illumina sequencing reads can be challenging. All three types of determinants are frequently plasmid-borne and often co-localize with transposons and other mobile genetic elements with multiple copies, leading to assembly gaps and localization near contig ends. To address this limitation, some studies reviewed in this section adopt a hybrid WGS approach, incorporating long-read sequencing to resolve the genetic context of these transferable elements. The long reads are typically generated using the MinION device from Oxford Nanopore Technologies, which offers excellent scalability and routinely produces reads exceeding 150 kilobases when high-molecular-weight DNA is used [109].

The following subsections will review all studies that have sequenced the genomes of clinical LRE isolates carrying at least one *cfr*, *optrA*, or *poxtA* gene.

#### 3.3.1. LNZ Resistance in Enterococci via Target Modification Mechanisms

LNZ resistance in enterococci, as well as in other Gram-positive pathogens can be manifested via alterations in the modifications of specific nucleotides in the 23S rRNA placed at or near an antibiotic binding site which can affect drug binding to the ribosome. While certain housekeeping modifications at the peptidyl transferase center have been shown to influence LNZ susceptibility, there is only one known so far that confers transferable LNZ resistance [55]. It is mediated by the multiresistance gene *cfr*, which encodes an rRNA methyltransferase [110]. This enzyme catalyzes the methylation of the C-8 position of the 23S rRNA nucleotide A2503, a key component of the LNZ binding pocket (Figure 1) [111]. This modification grants resistance to five distinct antibiotic classes that bind to overlapping but nonidentical sites within the peptidyl transferase center. The resulting phenotype is known as PhLOPS_A_, referring to resistance against phenicols, lincosamides, oxazolidinones, pleuromutilins, and streptogramin A antibiotics [55]. The Cfr protein has evolved from the housekeeping rRNA methyltransferase RlmN, highlighting how proteins related to core ribosomal compounds can serve as a crucial reservoir for resistance evolution due to their inherent ability to interact with and modulate the antibiotic’s target [106].

Several variants of *cfr* have been reported to date, including *cfr(B)*, *cfr(C)*, *cfr(D)*, and *cfr(E)* [112]. However, a recent study indicated that only *cfr(B)* and *cfr(D)* have been detected in enterococci through a search on NCBI PubMed [113]. Moreover, variants of the *cfr* gene have been infrequently identified among members of the genus *Enterococcus* in general [114]. Notably, nearly all reports associating LNZ resistance in enterococci with the *cfr* gene describe its presence in combination with other resistance determinants, such as *optrA*, *poxtA*, and/or mutations in the 23S rRNA gene. A widely cited case from Thailand describes a LREfs ST16 isolate recovered after prolonged treatment with the antibiotic. It is frequently presented as evidence implicating *cfr* in LNZ resistance in *E. faecalis*, as the isolate lacked mutations in the 23S rRNA gene and in the genes encoding ribosomal proteins L3 and L4 [115]. However, it is important to note that this report predates the identification of *optrA* and *poxtA*; therefore, their presence in the isolate cannot be definitively ruled out, particularly in the absence of WGS data necessary for retrospective resistome analysis. Furthermore, experiments involving the cloning and expression of *Cfr(D)* in *E. faecium* and *E. faecalis* did not confer any resistance, in contrast to similar experiments in *E. coli*, where the expected PhLOPS_A_ phenotype was observed [112]. The expression of the constructs was validated in all three cases using RT-qPCR. This observation highlights that, unlike 23S rRNA mutations, the presence of a *cfr* gene variant does not necessarily guarantee an LNZ-resistant phenotype in enterococci. Moreover, analyses of clinical and environmental isolates have shown that, even when the MIC is elevated, it is typically not as high as in cases with multiple G2576U variants. LRE strains with high levels of resistance often employ additional mechanisms alongside cfr-mediated methylation.

A summary of the data from WGS studies that have identified *cfr* gene variants in LRE is showcased in Table 4.

As evident from the summarized data, a total of 17 studies have reported the identification of *cfr* variants in sequenced LRE genomes. The geographical distribution is highly biased toward the Northern Hemisphere, with studies covering countries in Asia, Europe, and North America. Notably, only Denmark and Germany are represented by more than one study.

Significantly more *cfr*-positive LREfm isolates have been reported compared to LREfs. Overall, the number of sequenced genomes remains low. This can, to some extent, be attributed to the study designs—among all acquired LNZ resistance determinants, *cfr* was the only one not used as a selection criterion for LRE WGS in any of the identified studies with larger number of sequenced genomes. A similar explanation applies to the high prevalence of *cfr* + *optrA* or *cfr* + *poxtA* combinations observed in the analyzed LRE genomes.

The recent study by Cinthi et al. is of particular interest, as it describes two *E. faecium* and one *E. faecalis* isolate that were resistant to both LNZ and vancomycin due to the presence of *optrA*, *cfr(D)*, and *vanA* genes, all located on plasmids with a linear topology [52]. Notably, this study represents the first report of a linear plasmid in *E. faecalis* [52].

The highest number of described isolates (*n* = 7) originates from Pakistan, where the identified *cfr(D)* variants are accompanied by *poxtA* or, in one case, a combination of *poxtA* and *optrA* [46]. Given the presence of LRE isolates with G2576U mutations in Pakistan, as discussed in previous sections, the country emerges as a hotspot for LRE strains harboring diverse resistance mechanisms, including complex combinations of multiple determinants. This is likely attributable to Pakistan’s status as a high-burden country for multidrug-resistant tuberculosis, with one of the highest caseloads and incidence rates globally, as recognized by the WHO. The widespread use of LNZ in treatment regimens (e.g., bedaquiline, pretomanid, and LNZ, with or without moxifloxacin, in six-month all-oral therapies for Pakistan) has been instrumental in controlling tuberculosis. However, it has also contributed to a surge in LNZ resistance among enterococci.

Overall, the precise contribution of *cfr* gene variants to LNZ resistance in enterococci, if any, remains uncertain. Nevertheless, their presence in clinical *Enterococcus* isolates should not be neglected, as enterococci may serve as a reservoir for *cfr* and *cfr*-like genes, with the potential for horizontal transfer to staphylococcal species, where these determinants are clearly implicated in linezolid resistance [114].

#### 3.3.2. LNZ Resistance in Enterococci via Target Protection Mechanisms

The final mechanism of LNZ resistance identified in clinical Enterococci relies on target protection by members of the ATP-binding cassette (ABC)-F protein subfamily. These proteins bind to the ribosome, facilitating the release of ribosome-targeted antibiotics, thus rescuing the translation apparatus from antibiotic-mediated inhibition [119]. What is particularly remarkable is that the exact mechanism by which these proteins confer resistance was not clarified until 2016, after a debate that lasted over a quarter of a century. Moreover, this group is far from small or isolated. On the contrary, members of the ABC-F protein subfamily mediate resistance to a broader range of antibacterial drug classes than any other single group of resistance proteins. They constitute a major source of clinical resistance to nearly all antibacterial drug classes targeting the 50S subunit of the ribosome, including lincosamides, macrolides, oxazolidinones, phenicols, pleuromutilins, and streptogramins groups A and B [106]. Despite this, the precise mechanism of action was not resolved until 2016, bringing an end to a debate that had lasted for over 25 years [120].

ABC-F proteins differ from most other members of the ATP-binding cassette (ABC) superfamily in that they lack the typical transmembrane regions. Instead, they consist of two ABC domains separated by a linker region, which has been identified as the P site tRNA interaction domain, playing a key role in antibiotic resistance [121]. Based on their antibiotic specificity, three major categories of ARE ABC-F proteins can be distinguished: the first group mediates resistance to group A streptogramins, lincosamides, and occasionally pleuromutilins; the second group confers resistance to group B streptogramins and macrolide antibiotics (and sometimes ketolides); and the third group, including Optr and Poxt, mediates resistance to oxazolidinones and phenicols [106]. These will be further discussed in relation to their role in LNZ resistance in enterococci. The molecular mechanism by which Optr and Poxt proteins are able to dislodge oxazolidinones and phenicols from the ribosome requires further investigation since these proteins have relatively short antibiotic resistance domains, which would not typically be expected to extend into the peptidyl transferase center [122].

Although the precise molecular mechanism of target protection by OptrA and PoxtA remains to be fully elucidated, their role in conferring LNZ resistance in clinical LRE isolates is undeniable. The *optrA* gene encodes an ABC-F protein that targets the ribosome of Gram-positive bacteria, mediating resistance to both phenicols and oxazolidinones through ribosomal protection [106]. First identified in clinical isolates in 2015 [123], *optrA*-carrying LRE strains have since been detected in a diverse range of hosts, including both hospitalized and healthy humans [124].

Additionally, an *optrA* reservoir has emerged in animal populations, largely due to the widespread use of phenicols in veterinary medicine to treat respiratory infections [125]. OptrA-producing isolates exhibit considerable genetic diversity and are associated with various chromosomal and plasmid genetic platforms. Notably, *optrA* has been more frequently reported in *E. faecalis* than in *E. faecium* [124]. Given that recent meta-analyses indicate a rapid increase in the frequency of LREfs isolates in recent years, it is evident that *optrA* poses the greatest threat to the continued effectiveness of LNZ as a last-resort treatment for severe enterococcal infections [26,34].

Noteworthy, in addition to the wild-type *optrA* gene identified in numerous LRE isolates from diverse sources, at least 69 *optrA* variants have been described to date. These variants differ by 1 to 20 amino acid substitutions, and no less than 35 of them have been identified in *Enterococcus* species to date [114]. A recent study, conducted at a tertiary care hospital in Hangzhou (China), identified eight distinct *optrA* variants among 15 *optrA*-positive clinical isolates, demonstrating that such genetic diversity is also present in clinical settings [126]. Furthermore, data from multiple studies examining linezolid MIC values in various Gram-positive bacteria suggest a correlation between certain *optrA* variants and elevated LNZ-resistance levels. Variants such as the wild-type, D, EDP, KD, KLDP, RD, RDK, and RDKP are more frequently found in isolates exhibiting MIC values ≥ 8 mg/L [114].

Since its initial identification in an MRSA clinical isolate from Italy in 2018, *poxtA* has been detected in enterococci across multiple countries [127]. To date, it has been primarily reported in *E. faecium* isolates of human origin in Europe (Greece, Spain, Portugal, Ireland), as well as in the USA, Pakistan, and Turkey, with a prevalence among LREfm ranging from 16% to 78% [128]. Otherwise, *poxtA* is more commonly detected in environmental samples and food-producing animals than in human isolates, again with *E. faecium* exhibiting a higher prevalence than *E. faecalis* [128]. A variant of *poxtA*, designated *poxtA2*, was identified on a 13,746 bp plasmid in the linezolid-resistant *Enterococcus gallinarum* isolate Eg-IV02, recovered from a fecal swab of a healthy child in the rural Bolivian Chaco region. Hybrid whole-genome sequencing using both short and long reads revealed that *poxtA2* possesses a distinct genetic context compared to *poxtA* and likely represents its ancestor [129].

A summary of the data from WGS studies that have identified *optrA* gene and/or *poxtA* gene in LRE is showcased in Table 5.

A total of 42 studies from various countries worldwide have documented the global spread of *optrA* and *poxtA* among clinical LRE isolates. Notably, China leads with seven studies, followed by Spain with four. The most frequently detected isolate is LREfs carrying *optrA*, whereas *poxtA* is more commonly associated with LREfm, reinforcing previously observed biases in the distribution of these resistance determinants. Interestingly, a WGS-based study in the literature conducted its data processing before the identification of *optrA* and *poxtA*, failing to determine the LNZ resistance mechanism despite a clear resistant phenotype [146]. This highlights the potential value of reanalyzing previously sequenced genomes with unresolved resistance mechanisms, as it may lead to the discovery of previously unrecognized determinants or novel mutations.

The *optrA* and *poxtA* genes have been detected on both plasmids and chromosomal loci. Two studies even reported *optrA* detection on novel linear plasmids alongside *vanA* in both LREfm and LREfs isolates [51,52]. This finding is particularly concerning, as *optrA* dissemination is generally lower in *E. faecium* compared to *E. faecalis*, but the emergence of such novel vectors for horizontal gene transfer could alter this trend. Moreover, the co-presence of *vanA* further narrows the already limited treatment options.

An intriguing study from India employed a hybrid WGS approach using long Nanopore sequencing reads [84]. This analysis identified an LREfm isolate carrying two copies of *optrA*, one integrated into the chromosome and the other plasmid-borne, highlighting the potential of advanced NGS technologies in elucidating LNZ resistance mechanisms in *Enterococcus* spp. Similar findings for *poxtA* were reported by Lázaro-Perona et al., who identified a clinical LREfm isolate with an increased copy number of the plasmid carrying the *poxtA* gene [104]. Proteomic analysis confirmed the presence of the PoxtA and revealed elevated expression levels in the isolate.

Finally, the study by Hu et al. is particularly noteworthy, as it reports the first global identification and characterization of a clinical *E. gallinarum* strain harboring *poxtA* EF9F6 (LNZ MIC = 8 mg/L) [88]. Li et al. also describe a *optrA* KLPD-positive *Enterococcus hirae* isolate of human origin; however, their study was primarily designed to investigate *optrA* carriage among patients [126].

**Figure 3:**
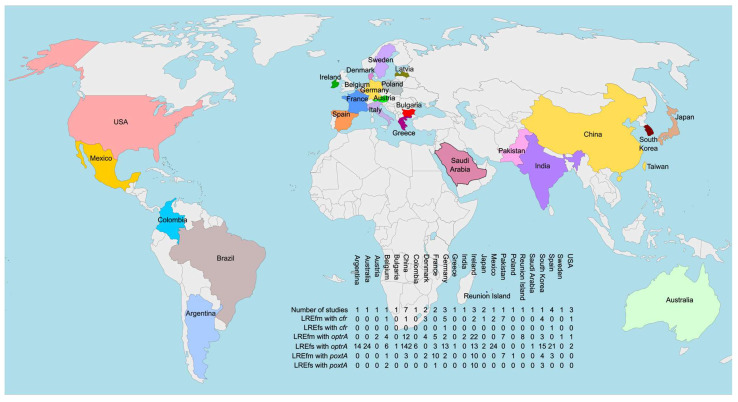
Geographical distribution of studies reporting sequenced genomes of linezolid-resistant *E. faecium* and/or *E. faecalis* strains harboring *cfr*, *optrA* or *poxtA* genes.

Figure 3 presents a consolidated map illustrating the geographical distribution of studies that have identified target protection resistance determinants in LRE.

### 3.4. Tedizolid—Resistance Mechanisms and Cross-Resistance with LNZ

Tedizolid (TDZ) is a second-generation oxazolidinone antibiotic developed after LNZ and approved in 2014 by the U.S. Food and Drug Administration for the treatment of acute bacterial skin and skin structure infections caused by susceptible Gram-positive bacteria [147]. Later, in 2015, TDZ was approved for medical use in the European Union by the European Medicines Agency [148]. It is administered as TDZ phosphate, a prodrug that is converted in vivo to the active compound, TDZ, through the action of phosphatases. This oxazolidinone represents a departure from the previously established structure–activity relationships described for LNZ. TDZ contains a hydroxymethyl side chain at the C-5 position, which was initially thought to reduce effectiveness [149]. However, this limitation was overcome by the incorporation of a fourth, para-oriented aromatic ring (the D-ring), which introduces additional hydrogen-bonding interactions and enhances stabilization of binding to the target site [150]. These structural modifications resulted in TDZ exhibiting strong in vitro activity against both MRSA and VRE. Moreover, it consistently demonstrated 4- to 8-fold greater potency compared to LNZ across a broad spectrum of Gram-positive microorganisms, including strains with reduced LNZ susceptibility [149,151].

According to The Surveillance of TDZ Activity and Resistance (STAR) program TDZ (MIC_50/90_, 0.25/0.25 mg/L; 99.9% susceptible) displayed activity similar to or greater than LNZ (MIC_50/90_, 1/2 mg/L; 99.5% susceptible), ampicillin (MIC_50/90_, 1/1 mg/L; 100.0% susceptible), daptomycin (MIC_50/90_, 0.5/1 mg/L; 99.6% susceptible), and vancomycin (MIC_50/90_, 1/2 mg/L; 98.1% susceptible) against a cohort of 4992 *E. faecalis* isolates [152]. Notably, in the same study TDZ demonstrated superior effectiveness against the vancomycin-resistant *E. faecalis* (VREfs) subset, showing 4- to 8-fold higher potency than LNZ (MIC_50/90_, 1/2 mg/L; 100.0% susceptible) and daptomycin (MIC_50/90_, 0.5/1 mg/L; 100.0% susceptible), respectively. In contrast, among the LNZ-non-susceptible *E. faecalis* isolates identified in the study, only 73.1% were inhibited by TDZ at concentrations ≤0.5 mg/L. Meanwhile, all of these isolates remained susceptible to ampicillin, daptomycin, and vancomycin at their respective clinical breakpoints, suggesting that TDZ and LNZ are likely to share certain resistance mechanisms. Furthermore, 6 of the 7 VREfs isolates exhibiting non-susceptibility to TDZ were found to be *optrA*-positive without accompanying 23S rRNA mutations, indicating a possible role of this determinant in the observed resistance phenotype [152]. Similar findings were reported in a recent study from Japan [153] in which three clinical LREfs isolates with TDZ MICs of 2–4 mg/L were also *optrA*-positive but lacked the G2576T mutation, whereas another three isolates harbored only this variant in two of their 23S rRNA loci and exhibited higher MICs of 8 mg/L. The analysis of 19 LREfm isolates from the same study revealed variable numbers of G2576T mutations across their genomes, with TDZ MICs ranging from 1 to 16 mg/L. As observed for LNZ, the number of affected 23S rRNA loci was not a reliable predictor of MIC values, since isolates with four mutated operons exhibited MICs of 2, 4, 8, and 16 mg/L, respectively, in the absence of other known LNZ-resistance determinants [153]. Conversely, the sole isolate harboring the T2504A variant in two of its 23S rRNA loci exhibited the highest observed MIC of 32 mg/L.

A large-scale study analyzing 916 *E. faecium* and 1342 *E. faecalis* isolates from invasive infections in hospitalized patients across U.S. and European medical centers (2015–2017) reported that TDZ inhibited all *E. faecalis* isolates except for one [151]. Among the *E. faecium* isolates, seven were LNZ-non-susceptible (MIC 4–8 mg/L), and five of these also exhibited TDZ nonsusceptibility (MIC ≥ 1 mg/L). Notably, one *E. faecium* isolate from Ankara, Turkey, demonstrated an elevated TDZ MIC of 0.5 mg/L despite lacking G2576T variants, while carrying both *optrA* and *poxtA*. This observation supports previous in vitro evidence indicating that *poxtA* can contribute to reduced susceptibility to TDZ [127].

One of the notable advantages of TDZ is that its MIC values appear largely unaffected by the presence of *cfr* genes, which have been implicated in several reported outbreaks of LNZ-resistant organisms [149]. However, this advantage is of limited relevance in LRE, where the role of this determinant in conferring LNZ resistance remains uncertain. The same applies to the Gly152Asp mutation of the 50S ribosomal protein L3, which has been found to solely confer cross-resistance to both LNZ and TDZ in MRSA isolates [154].

All findings on LNZ resistance mechanisms in enterococci and their impact on TDZ cross-resistance are summarized in Table 6.

### 3.5. Future Perspectives on WGS for Detecting and Investigating LNZ Resistance in Enterococci

With the decreasing costs of long-read NGS technologies such as Nanopore, new opportunities have emerged for studying LNZ resistance mechanisms associated with acquired genetic determinants. Notably, two of the most compelling discoveries, an LREfm isolate harboring two *optrA* copies in different genomic locations and another with an increased plasmid-borne *optrA* copy number, were made using sequencing data generated by a MinION device [84,104].

Moreover, a recent study by Coll et al. focused on evaluating and enhancing the accuracy of antibiotic resistance detection in *E. faecium* genomes for diagnostic and surveillance purposes [155]. The authors achieved near-complete genotype–phenotype concordance after re-evaluating false negatives, reporting a sensitivity of 100.0% [90.8–100.0] and a specificity of 98.3% [97.8–98.7]. Their findings align with the present literature review, where only one study entirely failed to identify the underlying LNZ resistance mechanism in the isolate described [146]. In a few other cases, individual isolates exhibited phenotypic LNZ resistance despite no identifiable resistance mechanism being detected through sequencing. However, such cases remain sporadic [105,156].

The study by Beh et al. aimed to elucidate the genomic epidemiology and population structure of LRE in Victoria, Australia, as well as at a global scale [157]. In a noteworthy shift in paradigm, the authors focused on isolates exhibiting phenotypic and/or genotypic resistance to linezolid, subjecting them to WGS to identify a range of resistance-associated determinants, including *cfr*, *cfr(B)*, *cfr(D)*, *optrA*, and *poxtA*. The resulting genomic data were compared with all publicly available sequencing reads and/or assemblies from comparable strains in the NCBI Pathogen Detection database and the Sequence Read Archive. These findings suggest that future research will likely emphasize genotypic characterization of LNZ resistance in enterococci, with epidemiological and population structure analyses increasingly conducted through the lens of modern bacterial genomics.

Another noteworthy application of NGS in diagnosing enterococcal infections is demonstrated in the work of Li et al. [158]. The authors present a metagenomic NGS (mNGS)-based approach for detecting E. faecalis in a urinary tract infection case. This innovative, culture-independent method proved to be rapid, sensitive, and highly accurate for pathogen identification while also providing valuable insights to guide clinicians in selecting an appropriate treatment. Additionally, mNGS enabled the assessment of treatment efficacy by comparing unique reads before and after LNZ administration. This suggests its potential for monitoring the patient’s microbiome for the occurrence of LRE, particularly in clinical indications requiring prolonged LNZ-based therapy.

## 4. Conclusions

The present review article emphasizes WGS as an indispensable tool for elucidating the molecular basis of LNZ resistance in enterococci, uncovering a complex interplay of resistance mechanisms. These include previously unreported 23S rRNA mutations, novel amino acid exchanges in ribosomal proteins of isolates lacking other known LNZ resistance determinants, and multiple copies of chromosomal- and plasmid-born ABC-F protein-encoding genes in single strains. Genomic analyses have shown that many LRE isolates harbor multiple resistance mechanisms, often in combination, particularly in geographical regions where LNZ is used in prolonged treatment courses for multi-drug-resistant tuberculosis. As LRE continue to evolve and acquire additional resistance traits, WGS-based research remains critical for tracking its dissemination, identifying novel resistance determinants and high-risk clones, and guiding antimicrobial stewardship efforts worldwide.

In the context of a growing pandemic of AMR, the emergence of LNZ resistance in clinical *Enterococcus* spp. isolates, especially in VRE, is a worrisome situation worldwide. There is no specific approved drug currently available for the treatment of infections due to those multidrug-resistant pathogens, although few studies have demonstrated the successful outcome using tigecycline and daptomycin separately [159]. Therefore, control of indiscriminate use of LNZ, robust global and regional surveillance studies on its resistance rates and mechanisms, as well as integration of antimicrobial stewardship programs into prevention strategies in hospital settings to limit the spread of LRE strains are necessary.

## Figures and Tables

**Table 1:** A compilation of studies utilizing whole-genome sequencing for the detection of 23S rRNA mutations associated with LNZ resistance in clinical *Enterococcus* spp. isolates.

Country	Year	Genomes with 23S rRNA Mutations	Additional Resistance Mechanisms	Source
Australia	2016–2021	1 LREfs with G2576U (3 loci affected)	No Data	[69]
Austria	2014–2017	13 LREfm with G2576U and 3 of them also harbor A2598G variant	No Data	[70]
Austria	2017	1 LREfs with G388A/D130N (1 locus), T2802 C/I934M (1 locus) and T2838C/- (2 loci); 1 LREfs with G388A/D130N (3 loci), T2802 C/I934M (1 locus) and T2838C/- (2 loci)	*optrA_4*, T301C/F101L in L4; *optrA_3*, T301C/F101L in L4	[71]
Belgium	2013–2025	7 LREfm with G2576U	1 LREFm with *cfr*	[49]
Brazil	2013	1 LREfm with G2576U	No Data	[72]
Bulgaria	2018	1 LREfs with G2576U (3 loci) 1 LREfs with C2163T (1 locus)	No Data *optrA*	[73]
China	-	1 LREfs with G2576U (2 loci)	No Data	[74]
China	2011–2015	16 LREfs with G2576U	10 LREfs with *optrA*	[75]
China	2018	2 LREfs with novel mutations	No Data	[76]
Czech Republic	2021	1 LREfm with G2576U	No Data	[77]
Denmark	2014–2023	52 LREfm with G2576U	1 LREfm with *cfr* + *poxtA*	[78]
Denmark	2015–2022	31 LREfm with G2576U	1 LREfm with *optrA*	[79]
France	2006–2016	4 LREfm with G2576U	3 LREfm with optrA	[80]
Germany	2014–2018	96 LREfm with G2576U	1 LREfm with *poxtA*	[81]
Germany	2007–2017	1 LREfs with G2576U	*optrA*	[82]
Germany	2013–2015	1 LREfm with G2576U (4 affected loci) 1 LREfm with G2576U (2 affected loci)	*cfr(B)* *cfr(B)*	[83]
India	2019	2 LREfm with G2592T	2 LREfm with *optrA*	[84]
Ireland	2016–2019	19 LREfm with G2576U (1–5 affected loci) 2 LREfs with G2576U	1 LREfm with *poxtA* No Data	[85]
Ireland	2013–2014	1 LREfm with G2576U (4 loci affected)	*optrA*, *cfr*, T150A in L3	[86]
Latvia	2021–2022	1 LREfm with G2576U	No Data	[87]
Pakistan	2021–2023	7 LREfm with G2576U	6 LREfm with *optrA*	[46]
South Korea	2019–2020	4 LREfm with G2576U	No Data	[88]
Spain	2017–2018	1 LREfs with G2576U	*optrA*	[89]
USA	-	3 LREfm with G2576U (2 × 3 loci affected and 1 × 2 loci affected)	No Data	[90]
USA	2012–2013	1 LREfm with G2576U (1 locus affected)	*cfr(B)*	[91]
USA	2009–2019	4 LREfm with G2576U	No Data	[92]
USA	2015	1 LREfm with G2576U (2–3 loci affected)	No Data	[93]
USA	2017	4 LREfm with G2576U	No Data	[47]
USA	2013–2016	2 LREfm with G2576U	No Data	[94]
USA	2018–2019	3 LREfs with G2576U	No Data	[51]
USA	2015–2016	29 LREfm with G2576U	No Data	[95]
ZAAPS programme	-	2 LREfm with G2576U	No Data	[96]

LNZ: linezolid; LREfm: linezolid-resistant *E. faecium*; LREfs: linezolid-resistant *E. faecalis*.

**Table 2:** LNZ-resistant *E. faecium* isolates from Ireland with different copy numbers of the G2576U mutation—Egan et al. [85].

Isolate	1	2	3	4	5	6	7	8	9	10	11	12	13	14	15	16	17	18
Sequence type	80	203	787	203	203	203	17	17	787	787	789	787	789	117	80	80	80	80
G2576 copy number	2	3	3	2	2	2	4	5	1	3	3	2	4	2	3	2	3	3
LNZ MIC (mg/L)	≥ 256	≥ 256	≥ 256	≥ 256	16	48	48	64	32	32	≥ 256	≥ 256	≥ 256	32	≥ 256	64	16	64

LNZ: linezolid; MIC: minimum inhibitory concentration.

**Table 3:** A compilation of studies utilizing whole-genome sequencing for the detection of missense mutations in the L3 and L4 ribosomal proteins associated with LNZ resistance in clinical *Enterococcus* spp. isolates.

Country	Year	Genomes with L3 and/or L4 Mutations	Additional Resistance Mechanisms	Source
Austria	2017	1 LREfm with T301C/F101L in L4 1 LREfm with T301C/F101L in L4 + C120T/- in L22	*optrA* + 23S mutations (see Table 1) *optrA* + 23S mutations (see Table 1)	[71]
China	2014–2018	1 LREfm with Ser77Thr in L22	No Data	[101]
Germany	2007–2017	13 LREfs with F101L in L4	*optrA*	[82]
Germany	2013–2015	2 LREfm with _211_GGT_213_ (glycine insertion) in L4	*cfr(B)*	[83]
Ireland	2013–2014	1 LREfm with T150A in L3	G2576U (4 loci affected), *optrA*, *cfr*	[86]
Italy	2016	1 LREfs with 71Gly72 in L4	No Data	[102]
Scotland	2014–2017	5 LREfs with T150A in L3 and F101L in L4 1 LREfs with T150A in L3 and F101L in L4	*optrA* *optrA* + *cfr(D)*	[103]
Spain	2023	1 LREfm with Lys68Glu in L4	*poxtA*	[104]
SENTRY Program	2008–2016	4 LREfs with F101L in L4 1 LREfs with F101L in L4	*optrA* *optrA* + *cfr*	[105]

LNZ: linezolid; LREfm: linezolid-resistant *E. faecium*; LREfs: linezolid-resistant *E. faecalis*.

**Table 4:** A compilation of studies utilizing whole-genome sequencing for the detection of *cfr* genes associated with LNZ resistance in clinical *Enterococcus* spp. isolates.

Country	Year	Genomes Harboring *cfr*	Additional Resistance Mechanisms	Source
Belgium	2013–2021	1 LREfm with *cfr(B)*	G2576U	[49]
China	2011–2022	1 LREfm with *cfr(D)*	*optrA*	[116]
Denmark	2014–2023	1 LREfm with *cfr* 1 LREfm with *cfr*	*poxtA* + G2576U *poxtA*	[78]
Denmark	2015–2022	1 LREfm with *cfrB*	No Data	[79]
Germany	2007–2017	1 LREfs with *cfr*	No Data	[82]
Germany	2013–2015	1 LREfm with *cfr(B)* 2 LREfm with *cfr(B)* 2 LREfm with *cfr(B)*	No Data G2576U _211_GGT_213_ insertion in the gene for L4	[83]
Ireland	2016–2019	1 LREfm with *cfr(D)*	*optrA*	[85]
Ireland	2013–2014	1 LREfm wit *cfr*	G2576U (4 copies), T150A in L3, *optrA*	[86]
Italy		2 LREfm with *cfr(D)* 1 LREfs with *cfr(D)*	*optrA* *optrA*	[52]
Japan	2017	1 LREfs with *cfr(B)*	*optrA*	[117]
Mexico	2023–2024	2 LREfs with *cfr(A)*	*optrA*	[118]
Pakistan	2021–2023	6 LREfm with *cfr(D)* 1 LREfm with *cfr(D)*	*poxtA* *optrA* + *poxtA*	[46]
Scotland	2014–2017	1 LREfs with *cfr(D)*	*optr(A)* + T150A in L3 + F101L in L4	[103]
South Korea	2019–2020	3 LREfm with *cfr(D)* 1 LREfm with *cfr(D)*	*poxtA* *optrA* + *poxtA*	[88]
Spain	2017–2018	1 LREfs with *cfr(D)*	*optrA*	[89]
USA	2012–2013	1 LREfm with *cfr(B)*	No data	[91]
SENTRY Program	2008–2016	1 LREfs with *cfr* 1 LREfs with *cfr*	*optrA* *optrA* + F101L in L4	[105]

LNZ: linezolid; LREfm: linezolid-resistant *E. faecium*; LREfs: linezolid-resistant *E. faecalis*.

**Table 5:** A compilation of studies utilizing whole-genome sequencing for the detection of *optrA* and *poxtA* genes associated with LNZ resistance in clinical *Enterococcus* spp. isolates.

Country	Year	Genomes Harboring *optrA*/*poxtA*	Additional Resistance Mechanisms	Source
Argentina	2016–2021	14 LREfs with *optrA*	No Data	[130]
Austarlia	2016–2021	24 LREfs with *optrA*	No Data	[69]
Austria	2017	2 LREfm with *optrA*	23S rRNA mutations (Table 1) and L4 mutations (Table 3)	[71]
Belgium	2013–2021	3 LREfm with *optrA* 1 LREfm with *optrA* and *poxtA* 61 LREfs with *optrA* 2 LREfs with *poxtA*	No Data No Data No Data No Data	[49]
Bulgaria	2023	1 LEEfs with *optrA*	23S rRNA mutation C2163T	[73]
China	2012–2021	11 LREfs with *optrA*	No Data	[131]
China	2014–2017	6 LREfm with *optrA* 1 LREfm with *optrA* + *poxtA* 1 LREfm with *poxtA*	No Data No Data No Data	[101]
China	2011–2022	2 LREfm with *optrA* 1 LREfm with *optrA* 1 LREfm with *poxtA* 61 LREfs with *optrA*	No Data *cfr(D)* No Data No Data	[116]
China	2009–2013	1 LREfm with *optrA* 12 LREfs with *optrA*	No Data No Data	[132]
China	2022–2023	5 LREfs with *optrA*	No Data	[133]
China	2011–2015	10 LREfs with *optrA* 13 LREfs with *optrA*	G2576U No Data	[75]
China	2018–2022	30 LREfs with *optrA*	No Data	[134]
Colombia	2014–2019	6 LREFs with *optrA*	No Data	[135]
Denmark	2014–2023	1 LREFm with *poxtA* 1 LREFm with *poxtA*	*cfr* *cfr* + G3576U	[78]
Denmark	2015–2022	3 LREfm with *optrA* 1 LREfm with *optrA*	No Data G2576U	[79]
France	2006–2016	3 LREfm with *optrA* 2 LREfm with *optrA* 3 LREfs with *optrA*	G2576U No Data No Data	[80]
France	2016–2020	10 LREfm with *poxtA* 1 LREfs with *poxtA*	No Data No Data	[128]
Germany	2014–2018	1 LREfm with *poxtA* 1 LREfm with *poxtA*	G2567U No Data	[81]
Germany	2007–2017	1 LREfm with *optrA* 1 LREfm with *optrA* 13 LREfs with *optrA*	No Data G2576U No Data	[82]
Greece	2018	1 LREfs with *optrA*	No Data	[136]
India	2019	1 LREfm with *optrA* 1LREfm with *optrA* 2 copies	G2592T G2592T	[84]
Ireland	2019	19 LREfm with *optrA*	No Data	[137]
Ireland	2016–2019	1 LREfm with *optrA* + *poxtA* 1 LREfm with *optrA* 1 LREfm with *optrA* 9LREfm with *poxtA* 13 LREfs with *optrA* 10 LREfs with *poxtA*	No Data *cfr(D)* No Data No Data No Data No Data	[85]
Ireland	2013–2014	1 LREfm with *optrA*	G2576U (4 copies), T150A in L3, *cfr*	[86]
Italy		2 LREfm with *optrA* 1 LREfs with *optrA*	*cfr(D)* *cfr(D)*	[52]
Japan	2017	1 LREfs with *optrA*	*cfr(B)*	[117]
Japan	2021	1 LREfs with *optrA*	No Data	[138]
Mexico	2023–2024	22 LREfs with *optrA* 2 LREfs with *optrA*	No Data *cfr(A)*	[118]
Pakistan	2021–2023	6 LREfm with *optrA* 1 LREfm with *optrA* + *poxtA* 6 LREfm with *poxtA*	G2576U *cfr(D)* *cfr(D)*	[46]
Poland	2020	1 LREfm with *poxtA*	No Data	[139]
Reunion Island	2015–2019	8LREfm with *optrA*	No data	[140]
Saudi Arabia	2014–2015	1 LREfs with *optrA*	No Data	[141]
Scotland	2014–2017	5 LREfs with *optrA* 1 LREfs with *optrA*	T150A in L3 + F101L in L4 *cfr(D)* + T150A in L3 + F101L in L4	[103]
South Korea	2017–2019	2 LREfs with *optrA*	No data	[142]
South Korea	2019–2020	2 LREfm with *optrA* 1 LREfm with *optrA* + *poxtA* 3 LREfm with *poxtA* 15 LREfs with *optrA* 3 LREfs with *poxtA*	No Data *cfr(D)* *cfr(D)* No Data No Data	[88]
Spain	2016–2017	5 LREfs with *optrA*	No Data	[143]
Spain	2017–2018	11 LREfs with *optrA* 1 LREfs with *optrA* 1 LREfs with *optrA*	No Data *cfr(D)* G2576U	[89]
Spain	2023	2 LREfm with *poxtA* 1 LREfm with *poxtA*	No Data Lys68Glu in L4	[104]
Spain	2018	3 LREfs with *optrA*	No Data	[144]
Sweden	2017–2020	1 LREfm with *optrA*	No Data	[145]
USA	2013–2016	1 LREfs with *optrA*	No Data	[94]
USA	2018–2019	1 LREfm with *optrA* 1 LREfs with *optrA*	No Data No Data	[51]
SENTRY Program	2008–2016	4 LREfs with *optrA* 1 LREfs with *optrA* 1 LREfs with *optrA*	F101L in L4 *cfr* + F101L *cfr*	[105]
ZAAPS Program	-	8 LREfs with *optrA*	No Data	[96]

LNZ: linezolid; LREfm: linezolid-resistant *E. faecium*; LREfs: linezolid-resistant *E. faecalis*.

**Table 6:** A compilation of LNZ resistance mechanisms detected in clinical *Enterococcus* spp. isolates.

Mechanisms	Involved Mutations/Determinants	Associated Phenotypes	Geographic Distribution of WGS Studies and Clinical Significance
Modifications of LNZ binding pocket	23S rRNA mutations; G2576U most frequent	LNZ MICs: 8– ≥ 256, generally higher with more 23S rRNA loci affected, but many exceptions; TDZ MICs: 4–8× lower, except T2504A (MIC 32)	Asia, Australia, Europe, North America, and South America; mainly LREfm; non-transferable
Modifications of LNZ binding pocket	L3, L4 and L22 ribosomal proteins	LNZ MICs ≤8 in most cases; TDZ—important in staphylococci	Asia, Europe; mainly LREfs; non-transferable
Target modification	*cfr* genes	Unclear significance in LRE; low LNZ MICs; TDZ unaffected, clinically important in staphylococci	Asia, Europe, North America; mainly LREfm; transferrable—reservoir for staphylococci
Ribosome protection	*optrA* and *poxtA* variants	Varying MICs dependant on the variant; TDZ MICs 4–8× lower	Global dissemination; mainly LREfs; easily transferrable (especially *optrA*); *optrA* is the most widespread LNZ resistance mechanism in clinical LREfs

LNZ: linezolid; TDZ: tedizolid; MIC: minimum inhibitory concentration; LREfm: linezolid-resistant *E. faecium*; LREfs: linezolid-resistant *E. faecalis*.

## Data Availability

No new data were created or analyzed in this study. Data sharing is not applicable to this article.
